# Advances in the nanostructure characterization of biological hydrogels formed by the prion-like domain of EARLY FLOWERING 3

**DOI:** 10.1107/S2059798326006698

**Published:** 2026-07-29

**Authors:** Stephanie Hutin, Pedro L. O. Filho, Anton M. Popov, Petra Pernot, Chloe Zubieta, Mark D. Tully

**Affiliations:** a Laboratoire Physiologie Cellulaire et Végétale, Université Grenoble Alpes, CNRS, CEA, INRAE, IRIG–DBSCI–LPCV, 17 Avenue des Martyrs, 38043Grenoble, France; b Materials Innovation Factory University of Liverpool 51 Oxford Street LiverpoolL7 3NY United Kingdom; c Structural Biology Group European Synchrotron Radiation Facility (ESRF) 17 Avenue des Martyrs 38043Grenoble France; d European Molecular Biology Laboratory, 17 Avenue des Martyrs, 38000Grenoble, France; University of Manchester, United Kingdom

**Keywords:** liquid–liquid phase separation, EARLY FLOWERING 3, SAXS, hydrogels, Gel-Cell

## Abstract

Hydrogels exhibit traits of both liquids and solids, forming a 3D structural network with generally high water content, high porosity and increasing stability over time. However, probing the structure of hydrogels is challenging due to the semi-disordered nature of their internal molecular organization and the presence of multiple species with varying dynamics. Small-angle X-ray scattering (SAXS) is well suited to study materials of this nature due to its ability to probe different length scales and to provide information as to the structure and conformation of different molecular species within the hydrogel. Here, we used the prion-like domain from the plant thermosensory protein EARLY FLOWERING 3 (ELF3) as a model hydrogel for structural characterization by SAXS. To overcome sample-handling challenges, we developed an X-ray- and vacuum-compatible sample cell for hydrogel manipulation that allows direct measurement in the X-ray beam. Furthermore, by using a three-component model, which gathers the scattering contributions from the different species in the hydrogel, which arise from individual and aggregated oligomers as well as from their stacking into a loose lamellar phase, we were able to satisfactorily fit the experimental data and provide a full description of the nanostructure of the ELF3 hydrogel.

## Abstract 2

In biological systems, hydrogels often arise through liquid–liquid phase separation, where biomolecular condensates can age into gel-like states. SAXS is a key technique to probe the molecular organization of these samples. This study introduces an in-vacuum Gel-Cell for SAXS-based structural analysis of a biologically derived hydrogel formed by the thermosensory prion-like domain of EARLY FLOWERING 3 and presents a three-component model to interpret the obtained data.

## 1. Introduction

Hydrogels exhibit important material characteristics including viscoelasticity, porosity, generally high solvent content, degradability and microarchitecture, among other properties that are distinct from liquids or solids (Cao *et al.*, 2021 ▸). Within the category of hydrogels, they can be further classified as either natural or synthetic, depending on the gelator involved (whether it is a naturally occurring polymer such as collagen from animals or a synthetic polymer such as polyacrylate, for example; Diaferia *et al.*, 2019 ▸). Based on their physical structure, hydrogels are further defined as amorphous, semicrystalline or crystalline materials with either covalently or physically cross-linked networks of molecules (Bustamante-Torres *et al.*, 2021 ▸). Hydrogel-forming polymers include biological macromolecules, such as proteins and polysaccharides, and are found as naturally occurring species in different organisms and cell types (Cao *et al.*, 2021 ▸).

In biological systems, liquid–liquid phase separation (LLPS) is often the first step and required for hydrogel formation, with many liquid condensates ageing into hydrogels both *in vitro* and *in vivo* (Wang *et al.*, 2021 ▸). A number of proteins that undergo LLPS and initially form highly dynamic liquid condensates may become more viscoelastic and rigid over time, transforming into gels or fibrils (Molliex *et al.*, 2015 ▸; Patel *et al.*, 2015 ▸; Ray *et al.*, 2020 ▸; Wegmann *et al.*, 2018 ▸; Murakami *et al.*, 2015 ▸). Fibril formation has been shown to be associated with neurological diseases and it has been suggested that phase separation increases the nucleation rate for protein aggregation into amyloid fibrils, aggregates and hydrogels (Pak *et al.*, 2016 ▸; Lin *et al.*, 2015 ▸; Xiang *et al.*, 2015 ▸). While fibril-forming proteins are relatively well studied due to their well defined structure, amorphous or semi-ordered hydrogels are more challenging to characterize and their internal structure is often poorly understood.

**Figure 1:**
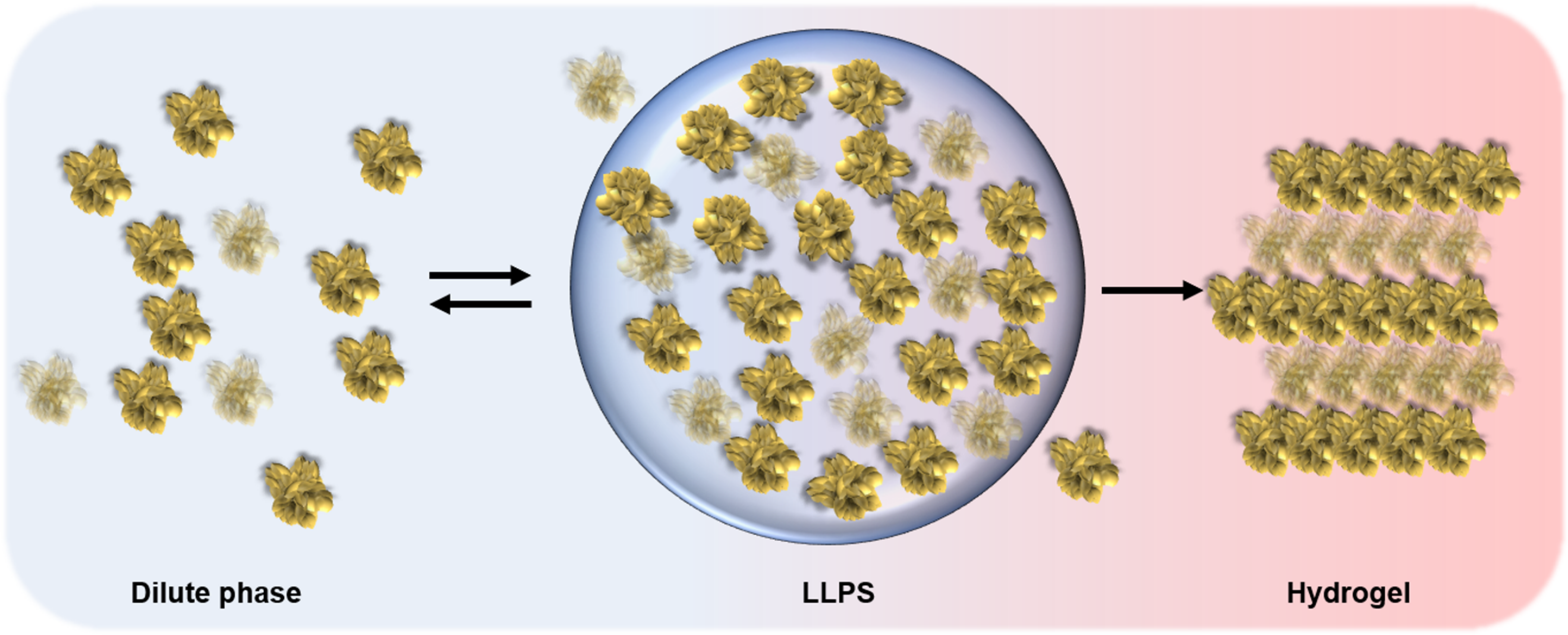
Scheme showing the formation of the hydrogel from the ELF3 PrLD domain.

To address this, we have investigated hydrogel formation by the prion-like domain (PrLD) of the *Arabidopsis thaliana* scaffold protein EARLY FLOWERING 3 (ELF3), a domain that acts as a direct thermosensor through the formation of biomolecular condensates (Jung *et al.*, 2020 ▸; Fig. 1 ▸). The PrLD of ELF3 undergoes phase separation as a function of increasing temperature, converting from a dispersed to a liquid condensed and finally a hydrogel phase in a highly reproducible manner, making it a useful model for temperature-controlled SAXS experiments. We have previously used SAXS, in combination with mass photometry and atomic force microscopy (AFM), to show that in the native state three variants of the ELF3 PrLD domain form homogeneous oligomeric species of ∼30 copies which adopt a globular structure; this oligomerization is required for LLPS and hydrogel formation (Hutin *et al.*, 2023 ▸; Fig. 1 ▸). Variants of ELF3 PrLD are present in different naturally occurring accessions that differ primarily in the length of a polyglutamine repeat (polyQ) from seven to 29 glutamines across 181 natural *Arabidopsis* accessions and exhibit slight alterations in LLPS and hydrogel formation (Undurraga *et al.*, 2012 ▸; Tajima *et al.*, 2007 ▸). These variants provide a physiologically relevant set of proteins for characterization.

## 2. Methods

### 2.1. Expression of ELF3

ELF3 PrLD with either zero, seven or 20 glutamine residues (Q0, Q7 and Q20) in the polyQ variable motif (residues 388–625, AT2G25930, *Arabidopsis thaliana* ecotype Columbia, numbering based on the Q7 Columbia wild-type protein) was overexpressed in *Escherichia coli* BL21-CodonPlus-RIL cells (Novagen) at 18°C and purified as described previously (Jung *et al.*, 2020 ▸; Hutin *et al.*, 2023 ▸). Briefly, the cells were resuspended in lysis buffer [100 m*M* bis-Tris propane pH 9.4, 300 m*M* NaCl, 20 m*M* imidazole, 1 m*M* tris(2-carboxyethyl)phosphine (TCEP), EDTA-free protease inhibitors (ThermoFisher)] and lysed by sonication. The proteins were purified using a 1 ml Ni–NTA column washed with 50 column volumes (CV) of lysis buffer, 50 CV of high-salt buffer (100 m*M* bis-Tris propane pH 9.4, 1 *M* NaCl, 20 m*M* imidazole, 1 m*M* TCEP). They were eluted in 500 µl steps in 100 m*M* bis-Tris propane pH 9.4, 300 m*M* NaCl, 300 m*M* imidazole, 1 m*M* TCEP. The fractions of interest were determined by SDS–PAGE, pooled and dialysed for ∼2 h at 4°C in 50 m*M* bis-Tris propane pH 9.4, 500 m*M* NaCl, 1 m*M* TCEP. To maximize gel formation the proteins were then dialysed against 50 m*M* bis-Tris propane pH 7.8, 250 m*M* NaCl, 1 m*M* TCEP at a concentration of ∼4 mg ml^−1^ for 3 h, after which time the protein formed an amorphous gel.

### 2.2. Hydrogel SAXS measurements

The ELF3 PrLD hydrogel was sandwiched into the Gel-Cell with ∼30 µm thick Kapton tape. The Gel-Cell was mounted onto an *xyz* stage within a custom-designed vacuum chamber on beamline BM29 at the ESRF (Tully *et al.*, 2023 ▸). Ten frames of 1 s were collected on a PILATUS3 2M detector (Dectris) at a sample-to-detector distance of 2.83 m. Kapton tape and trapped air was first measured as a background followed by ∼2 µl of each hydrogel, pipetted using a cut-down pipette tip to help with insertion of the sample into the Gel-Cell. Data integration was carried out by an automated pipeline, *FreeSAS* (Kieffer *et al.*, 2022 ▸). Individual frames were checked for radiation damage with only similar frames being averaged, the empty cell was subtracted from the sample and initial analysis was performed using the software *Scatter IV* (Tully *et al.*, 2021 ▸). Figures were produced in *PRIMUS* (Franke *et al.*, 2025 ▸).

### 2.3. Computational methods

Equation (1) was implemented in Python. The fittings of experimental data were performed through a nonlinear least-squares minimization routine (‘Curve_Fit’) included in the package *Optimize* within the SciPy library (Virtanen *et al.*, 2020 ▸). Testing of fitting parameters is further detailed in the supporting information.

## 3. Results

In a previous investigation to study condensation processes (Hutin *et al.*, 2023 ▸), LLPS was induced for three ELF3 PrLDs (containing polyglutamine stretches of zero, seven and 20 residues, referred to as Q0, Q7 and Q20, respectively) via an increase in temperature from 4 to 27°C, which is a range that can be sampled directly during SAXS data collection. Interestingly, the scattering curves collected over this temperature range showed a peak that appeared in the low-*q* region upon LLPS and increased in magnitude with increasing temperature. The liquid condensate formation was initially reversible if the temperature was quickly decreased (Hutin *et al.*, 2023 ▸). However, after ageing at higher temperatures, the condensates coalesced macroscopically into hydrogels. While monodispersed samples and liquid condensed phases are amenable to SAXS measurements using an automated sample changer attached to a quartz capillary flow cell, this experimental setup is limited to liquid samples of low-to-medium viscosity (Tully *et al.*, 2023 ▸). However, due to the high viscosity of hydrogels, this experimental setup is inadequate for sample handling and measurement. The traditional approach in this case would be to measure the samples in air. This is not optimal due to the increased background created by the air scattering and extra vacuum windows which may hinder the measurement of important features related to the nanostructure under investigation, particularly if these are at high *q* values.

**Figure 2:**
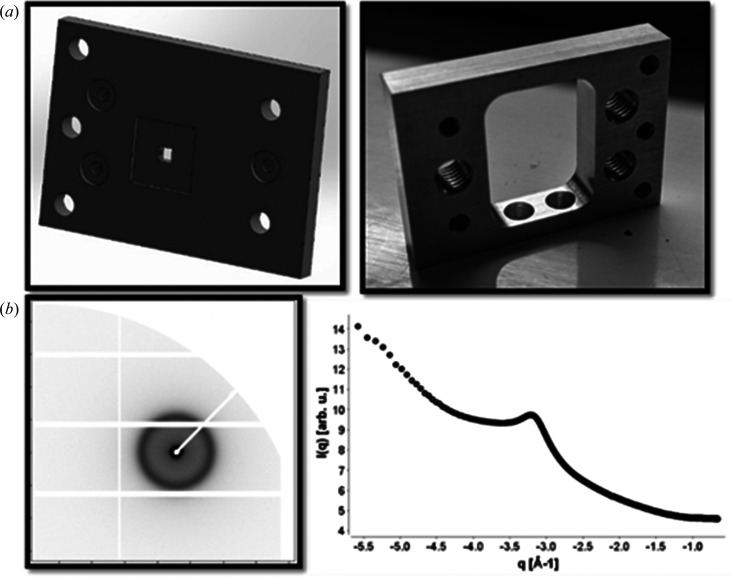
(*a*) New Gel-Cell and sample holder for gel-like samples designed at the BM29 SAXS beamline at the ESRF. (*b*) Detector image and corresponding subtracted curve for ELF3 Q0.

In this context, we developed a new X-ray-compatible vacuum cell, called the Gel-Cell, which allows the measurement of gel-like samples in vacuum. The cell consists of a 3D-printed device mounted on an aluminium holder. The (hydro)gel sample is sandwiched between two sheets of Kapton (or any other X-ray-transparent window material, such as mica or silicon nitride) and directly measured in vacuum (Fig. 2 ▸*a*). The Gel-Cell is compatible with the BioSAXS BM29 beamline at the ESRF and is adaptable to similar synchrotron SAXS beamline configurations. The dimensions of the Gel-Cell are described in Supplementary Table S1.

The Gel-Cell was used to measure the hydrogels formed by the Q0, Q7 and Q20 variants of the ELF3 PrLD investigated in a previous study (Hutin *et al.*, 2023 ▸). The obtained SAXS data (filled circles) demonstrated a prominent peak at the same *q* value as in the corresponding LLPS samples (Hutin *et al.*, 2023 ▸), but interestingly this peak was more intense (Figs. 2 ▸*b* and 3 ▸). The increase in the magnitude of the structure peak in the hydrogel samples indicates an increase in the ordering of the condensates during ageing. According to AFM and transmission electron microscopy (TEM) data from the ELF3 hydrogels, such a peak can be related to a lamellar stacking within the condensed phases (Hutin *et al.*, 2023 ▸). It should be noted that this layered arrangement is distinct from the characteristic fibril formation observed for amyloids (Shirahama & Cohen, 1965 ▸) and is less compact compared with the lamellar stacking that is typically observed for lipid membranes and polymer lamellae (Meisburger *et al.*, 2013 ▸; Hope *et al.*, 1985 ▸), which explains the existence of a relatively broad peak and the lack of higher order Bragg reflections.

**Figure 3:**
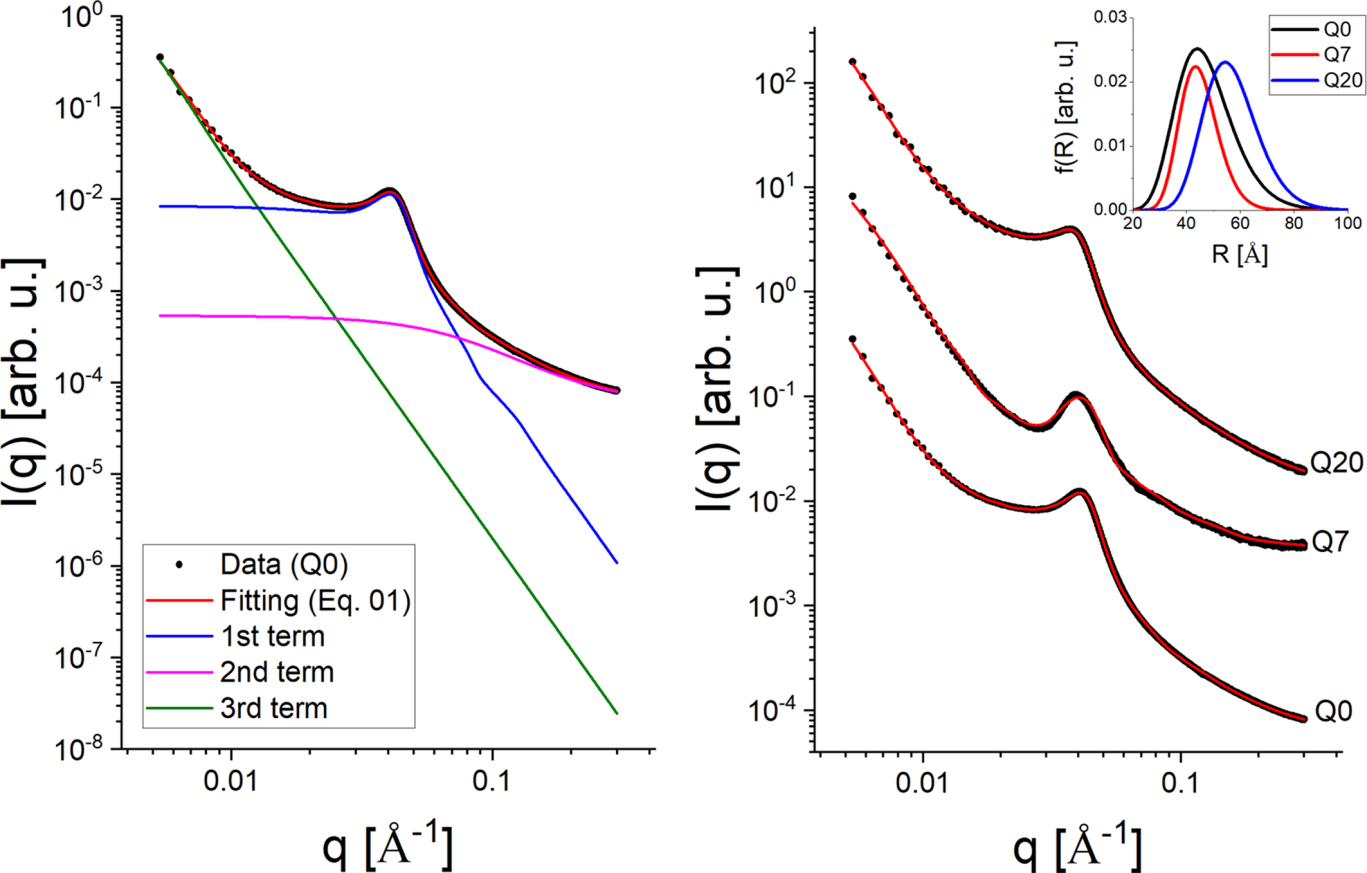
Left: visualization of each term forming equation (1) used to fit the SAXS data (black filled circles). The second term also includes the constant background back. Right: SAXS data of samples Q0, Q7 and Q20, fitted with equation (1) (red continuous lines). Inset: size distributions of the particles obtained from the fittings.

A simplistic quantitative analysis of the scattering curves presented in Fig. 3 ▸ would evaluate the position of the peaks assumed to be the first Bragg reflection of a lamellar phase (Miller index 100), and from this one can calculate the lattice factor *a* (also known as lamellar periodicity) through *a* = 2π/*q*_100_. Aiming to estimate the uncertainty of *a*, the peaks may be fitted with a peak function (*e.g.* Gaussian, Lorentzian, Voigt *etc.*) and the uncertainty of *q*_100_ may then be propagated to *a*. Additionally, a sloping background may also be introduced, in combination with the peak function, to locally fit the curve and again extract the *q*_100_ value. Although these techniques are useful and convenient, such an approach is limited and unable to retrieve or derive further information about the objects forming the lamellar arrangement (*i.e.* large oligomers) and, as such, it is impossible to establish a direct structural comparison with the data from the dilute and liquid condensed phases reported previously (Hutin *et al.*, 2023 ▸).

To address these shortcomings in data analysis, we propose a simple three-term model that gathers all of the essential physical information on the investigated system and represents the scattering from all of the different species present in the sample: individual oligomers, aggregated oligomers and stacked layers. In this model, the theoretical scattered intensity is expressed by
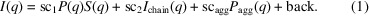
The first term reflects the use of the decoupling approximation (Kotlarchyk & Chen, 1983 ▸) to factorize the form factor *P*(*q*), describing the size and shape of the multimeric assembly of the ELF3 PrLD sequences (the 30-mer oligomer ‘building block’), and the structure factor *S*(*q*), describing the lamellar stacking showed by previous AFM and TEM experiments on the hydrogels (Hutin *et al.*, 2023 ▸). Aiming to be as generic as possible while keeping the number of fitting parameters as low as possible, the oligomeric assembly is represented in our model by polydisperse spheres, whose form factor is given by (Pedersen, 1997 ▸)
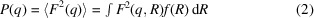
with
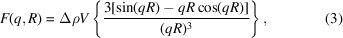
where *R* is the radius of the sphere with volume *V* and contrast scattering length Δρ relative to the medium where the object is located. The number size distribution, *f*(*R*), is arbitrarily evaluated in our case by a lognormal function (Losito *et al.*, 2021 ▸). Other more complex functions can be used to describe the globular oligomers, such as the revolution and triaxial ellipsoidal form factors (Kotlarchyk & Chen, 1983 ▸), which require extra fitting parameters. Nevertheless, since the overall fitting quality was not improved in our case, we continued using the form factor given by equation (2).

The contribution of the lamellar stacking to the total scattering can be analytically represented by Förster *et al.* (2005 ▸),
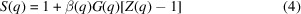
with
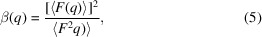

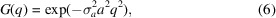

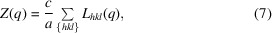

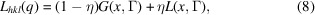
where *a* is the lattice factor, defined above. The parameter σ_*a*_ in the Debye–Waller factor (equation 6) quantifies the distortion relative to an ideal lamellar lattice and, in combination with β(*q*) (equation 5), reduces the higher order Bragg reflections (Freiberger & Glatter, 2006 ▸), whereas *c*, appearing in equation (7), ensures that the product of the form factor and structure factor fulfils the equation for the Porod invariant (Förster *et al.*, 2005 ▸). One practical advantage of the chosen structure factor, among other possible options (Nallet *et al.*, 1993 ▸; Zhang *et al.*, 1994 ▸), is related to the freedom of defining the peak profile *L*_*hkl*_(*q*) (equation 8), given in our case by a pseudo-Voigt function (Losito *et al.*, 2021 ▸), *i.e.* a linear combination of Gaussian and Lorentzian functions that share the same full-width at half-maximum (FWHM), Γ, weighted by the parameter η, that varies between 0 and 1.

The second term in equation (1) is the contribution of a polymer-like scattering due to disordered polypeptide domains, modelled as a Gaussian chain with radius of gyration *R*_g_ (Sundblom *et al.*, 2009 ▸):
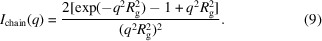
The third term describes the power-law slope of the scattering curve at low *q* values associated with the presence of oligomer aggregates. For simplicity, we used the same form factor given by equation (2) but with different *R* and σ_*R*_ (relative polydispersity). Finally, the parameters sc_1_, sc_2_ and sc_agg_ are scale factors, while back introduces corrections to the incoherent constant background (equation 1). A summary of the fitting parameters as well as their description is presented in Table 1 ▸, while a visualization of each term of equation (1) is shown in Fig. 3 ▸ (left). Note that because the fittings are performed on a relative scale, the constant Δρ appearing in equation (3) can be incorporated into sc_1_.

We used equation (1) to fit the SAXS curves shown in Fig. 3 ▸ (right). The fittings are represented by red continuous lines and, as shown by the SAXS data in black, the model satisfactorily describes the scattering profile over all of the probed *q* range. The obtained values for the fitting parameters are summarized in Table 1 ▸.

Overall, the nanostructure of the hydrogels is quite similar in all investigated samples, with a lamellar periodicity of 146 < *a* < 158 Å and a radius of the particles of 45 < *R* < 57 Å. As expected, due to its longer polypeptide chain, Q20 formed particles which showed a slightly larger size and lamellar periodicity. Relatively large values of σ_*a*_ (between 0.07 and 0.12 Å) are compatible with the observed short-range ordering of the system. Equally, larger aggregate sizes are observed (*R*_agg_ ≃ 620 Å) and an increase in the radius of gyration (*R*_g_ ≃ 130 Å) for Q20 relative to the other sequences which is, again, likely to be a direct consequence of the longer sequence. Moreover, although all the particles have moderate polydispersity, between approximately 16% and 23%, the aggregates are quite polydisperse (between 48% and 60%), indicating large heterogeneities in the samples due to aggregation.

Previous work reported that the Q0, Q7 and Q20 ELF3 particles in the dilute phase have radii of gyration of 72.4, 73.4 and 75.6 Å, respectively (Hutin *et al.*, 2023 ▸). Using the relation between these measured values and the corresponding radius of a sphere (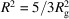), we obtain values of 93.5, 94.8 and 97.6 Å, almost the double of the *R* values presented in Table 1 ▸, This indicates that the particles in the hydrogel phase are smaller than those in the diluted phase, suggesting a compaction event of the oligomeric building blocks during gelation. In contrast, when considering the radii of gyration evaluated for the condensed phase (118, 128 and 134 Å), presented in the previous work (Hutin *et al.*, 2023 ▸), we obtain a corresponding sphere radius (of the aggregates) of 305, 330 and 346 Å, which is in fair agreement with the values of *R*_agg_ presented in Table 1 ▸. The lamellar periodicity values for hydrogels are 146, 146 and 157 Å for Q0, Q7 and Q20, respectively, and are in satisfactory agreement with the values obtained for the corresponding condensed phases (155, 163 and 167 Å for Q0, Q7 and Q20, respectively; Hutin *et al.*, 2023 ▸), although systematically smaller. The difference could suggest the existence of a slightly more compact lamellar phase in the hydrogel, similar to the microenvironments previously observed with AFM (Hutin *et al.*, 2023 ▸). Concurrently, the stacking spacing (*i.e.* the spacing between two adjacent layers) for the hydrogel phase, assessed by TEM, was found to be between 40 and 50 Å (Hutin *et al.*, 2023 ▸), which is in satisfactory agreement with the stacking distances obtained here: 51.3, 56.3 and 44.6 Å for Q0, Q7 and Q20, respectively.

## 4. Conclusion

In conclusion, we designed and tested a new sample holder that allows the measurement of gel-like samples in vacuum, greatly reducing the background scattering due to air scattering, which is crucial for low-scattering samples, such as protein hydrogels, especially when measured on a synchrotron beamline. The Gel-Cell is currently available at the BM29 SAXS beamline at the ESRF and is available for integration into other synchrotron beamlines of similar configuration. Secondly, we developed a simple three-component model (equation 1) that allows the investigation of the nanostructure of hydrogels, and present an experimental test case with variants of the prion-like domain of ELF3. The obtained information is satisfactorily corroborated by previous TEM and AFM analyses on the same hydrogels (Hutin *et al.*, 2023 ▸), which demonstrates the reliability of the proposed model and allows direct comparison with the structures present in the corresponding dilute and condensed phases previously investigated (Hutin *et al.*, 2023 ▸). From this, we concluded that the ELF3 PrD hydrogels are formed by more compact oligomeric globular structures (relative to the oligomers present in the dilute phase) and their nanostructure is very similar to those existing in the liquid condensed phase, although the loose lamellar phase of hydrogels is slightly more compact. The same model can in principle be used for the investigation of the nanostructure of any proteinaceous or polymer system where structure forms in the condensed phase, thus providing a general framework for studies of biological hydrogel structure. As always, it is important to verify whether the assumptions underlying equation (1) are satisfied, as they define the limits of the model’s applicability. If any assumptions are not met, equation (1) can be modified accordingly to accommodate the new conditions.

## Supplementary Material

Supplementary Table S1 and Supplementary Methods. DOI: 10.1107/S2059798326006698/he5695sup1.pdf

SAXS data.: https://doi.org/10.15151/esrf-dc-2490092768

## Figures and Tables

**Table 1:** Fitting parameters, their description and the obtained values for each investigated sample Uncertainties in the last digit are given in parentheses.

Parameter	Description	Q0	Q7	Q20
*a* (Å)	Lattice factor or, in this case, lamellar periodicity	146.3 (3)	146.1 (6)	157.6 (3)
σ_*a*_ (Å)	Quantifies the distortion relative to an ideal lamellar lattice, being zero for an ideal lattice	0.106 (6)	0.120 (9)	0.070 (6)
Γ	Full-width at half-maximum (FWHM) of the peak, the same for all peaks in the SAXS curve	0.0138 (1)	0.0140 (2)	0.0156 (2)
η	Varying from 0 to 1, the fraction of Lorentz function in the pseudo-Voigt function	0.21 (4)	1.00 (9)	0.07 (3)
*c*	Constant ensuring that the product of form factor and structure factor fulfils the equation for the Porod invariant	10 (1)	10 (8)	10 (1)
*R* (Å)	Radius of the particles	47.5 (3)	44.9 (7)	56.5 (1)
σ_*R*_ (%)	Relative polydispersity of *R*	23 (4)	16 (4)	18 (1)
*R*_g_ (Å)	Radius of gyration related to the polymer-like scattering due to the disordered domains	21 (1)	32 (9)	122 (8)
*R*_agg_ (Å)	Radius of the aggregates	375 (11)	237 (5)	334 (9)
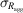 (%)	Relative polydispersity of *R*_agg_	48 (1)	60 (1)	50 (1)
sc_1_	Scale factor	0.056 (3)	0.009 (7)	0.037 (1)
sc_2_	Scale factor	0.0013 (1)	0.003 (2)	0.026 (3)
sc_agg_	Scale factor	0.92 (1)	0.93 (1)	0.94 (1)
Back × 10^−6^	Constant incoherent scattering contribution	158 (1)	372 (1)	86 (1)

## Data Availability

SAXS data are available at https://doi.org/10.15151/esrf-dc-2490092768 and Python scripts are available upon request.
