# Supplementary material for: Advances in the nanostructure characterization of biological hydrogels formed by the prion-like domain of EARLY FLOWERING 3
Source: Acta Crystallogr D Struct Biol. 2026 Jul 29;82(Pt 8):940–6. doi: 10.1107/S2059798326006698 (PMC13431640; doi:10.1107/S2059798326006698)
Supplement: Supplementary file 1 [file d-82-00940-sup1.pdf]

Acta Cryst D

STRUCTURAL BIOLOGY

Volume 82 (2026)

Supporting information for article:

Advances in the nanostructure characterization of biological hydrogels formed by the prion-like domain of EARLY FLOWERING 3

Stephanie Hutin, Pedro L. O. Filho, Anton M. Popov, Petra Pernot, Chloe Zubieta and Mark D. Tully

Table S1 SAXS Data-collection parameters

SAXS Data-collection parameters	
Instrument:	ESRF BM29
Wavelength (Å)	0.99
q-range ($Å^{-1}$)	0.007-0.5
Sample-to-detector distance (m)	2.81
Concentration range (mg/mL)	1-6
Temperature (K)	293
Detector	Pilatus P3-2M
Flux (photons/s)	$1.4*10^{12}/1*10^{13}$
Beam size at sample (µm)	500*200
**Gel-Cell Dimensions**	
Device Size (mm)	35 x 25
Size of Opening (mm)	1.2 x 1.2
Path Length (mm)	0.8
Max Sample Volume (mm^3)	1.152
**Software employed**	
Primary data reduction:	FreeSAXS,
Data processing	ScatterIV, python Script, Optimize, Primus

S1. Methods 1

S1.1. Fitting Parameters

To assess the reliability of the fitted parameters, we analysed the covariance matrix returned by the nonlinear least-squares routine (scipy.optimize.curve_fit), focusing on the associated standard uncertainties for each parameter. In all cases, the resulting uncertainties are small compared to the parameter values, indicating that the fitted parameter set is well constrained by the data and that the model is not numerically ill-conditioned.

We further evaluated the robustness of the optimisation by performing fits starting from multiple, widely different sets of initial parameter values. In all cases, the procedure converged reproducibly to the same solution within uncertainty, which supports the stability of the optimisation and the robustness of the reported parameters. In addition, we imposed physically motivated bounds on all parameters, ensuring that the optimisation explores only a realistic region of parameter space and avoiding unphysical compensations between parameters.
